# Mechanical response of cardiovascular stents under vascular dynamic bending

**DOI:** 10.1186/s12938-016-0135-8

**Published:** 2016-02-20

**Authors:** Jiang Xu, Jie Yang, Nan Huang, Christopher Uhl, Yihua Zhou, Yaling Liu

**Affiliations:** School of Mechanics and Engineering, Southwest Jiaotong University, 610031 Chengdu, People’s Republic of China; School of Material Engineering and Science, Southwest Jiaotong University, 610031 Chengdu, People’s Republic of China; Bioengineering Program, Lehigh University, Bethlehem, PA 18015 USA; Department of Mechanical Engineering and Mechanics, Lehigh University, Bethlehem, PA 18015 USA

**Keywords:** Cardiovascular stent, Vascular dynamic bending (VDB), Vascular pulsation (VP), Stent fatigue, Explicit-implicit coupling simulation method

## Abstract

**Backround:**

Currently, the effect of vascular dynamic bending (VDB) has not been fully considered when studying cardiovascular stents’ long-term mechanical properties, as the previous studies about stent’s mechanical properties mostly focus on the effect of vascular pulsation (VP). More and more clinical reports suggested that the effect of VDB have a significant impact on stent.

**Methods:**

In this paper, an explicit-implicit coupling simulation method was applied to analyze the mechanical responses of cardiovascular stents considering the effect of VDB. The effect of VP on stent mechanical properties was also studied and compared to the effect of VDB.

**Results:**

The results showed that the dynamic bending deformation occurred in stents due to the effect of VDB. The effects of VDB and VP resulted in alternating stress states of the stent, while the VDB alternate stresses effective on the stent were almost three times larger than that of the VP. The stress concentration under VDB mainly occurred in bridge struts and the maximal stress was located in the middle loops of the stent. However, the stress distributed uniformly in the stents under the effect of VP. Stent fracture occurred more frequently as a result of VDB with the predicted fracture position located in the bridging struts of the stent. These results are consistent with the reported data in clinical literatures. The stress of the vessel under VDB was higher, than that caused by VP.

**Conclusions:**

The results showed that the effect of VDB has a significant impact on the stent’s stress distribution, fatigue performance and overall stress on the vessel, thus it is necessary to be considered when analyzing stent’s long-term mechanical properties. Meanwhile, the results showed that the explicit-implicit coupling simulation can be applied to analyze stent mechanical properties.

## Background

Vascular stenting, with its advantages of slight trauma and effective treatment, has been widely used in clinic [1]. The mechanical behavior of a coronary stent in the human body affects short-term and long-term therapeutic effects of vascular stenting [2, 3]. Thus, analyzing stent mechanical properties is of great importance for further improvement of stent design and effective treatment.

Currently, finite element method (FEM) is widely used to study stents’ mechanical properties. FEM studies about the stent solid mechanical properties are mainly categorized into two types: the first investigates mechanical properties of the stent during the process of free expansion without any external constraints; the other studies mechanical properties of stents surrounded by blood vessels. The first type is mainly applied to obtain the stent’s expanding pressure, axial contraction, flexibility, radial recoil rate, strain distribution in stent ends, uniformity of expansion, the “dog bone” phenomenon, fatigue of the stent and so on [4–12]. Its main purpose is to provide guidance for stent design and optimization. The second type is mainly for studying the interaction between the stent and the vessel. It aims to understand the clinical complications of stent implantation [i.e. in-stent restenosis (ISR) and stent fracture (SF)], while also providing technical support for stent surgery optimization [13–28].

Many clinical results have shown that stent fracture is one of the most important factors for serious complications (i.e. ISR) after stent implantation. Most stent fractures happened in the mid-late service time, although a few of them immediately fractured after implantation. Nakazawa et al. [29] found that stent complications occurred in 78 % of serious stent fracture cases. The statistical data from Umeda et al. [30] showed the restenosis rate increased from 3 to 15 % after stent fracture. The restenosis rate before and after stent fracture from Aoki et al. [31] was 12.4 and 37.5 % respectively. Lee et al. [32] claimed that stent restenosis was more likely to happen after stent fracture, because the fractured stent promoted neointimal or smooth muscle hyperplasia.

The fatigue of coronary artery stents is mainly caused by contractions of the heart (the systolic and diastolic function) where the main effects on the stent’s mechanical properties are the pulsation of vessels and vascular movement. Currently, the pulsation force of the vasculature has been applied to study the stent fatigue. Marrey et al. [33] investigated the development of micro cracks in stents and predicted the life of stents under the vascular pulse conditions based on fracture mechanics. Li et al. [34] analyzed the pulse pressure applied on stents by computational fluid dynamics. Making use of computational fluid dynamics, the pulsation force was calculated and then converted into a stress by the group. The stress was then used in conjunction with the S–N curve for the material in order to determine the expected lifespan of the stent in terms of cycles. Zhi et al. [35] studied the fatigue life of NiTi stents, which simplified the pulse pressure to a harmonic load on the inner surface of the stents. Others also analyzed the coronary stent’s fatigue performance, such as Weiß et al. [36] tested the coronary stent’s fatigue performance based on the stent test standard, H.A.F. Argente et al. [37] studied a balloon stent’s fatigue life based on a two-scale continuum damage mechanics model. H. M. Hsiao et al. [38, 39] studied the fatigue properties of renal artery stent in which the effect of VP and VDB was considered. Among these studies about cardiovascular stent’s mechanical performance only the effect of VP was being considered, the effect of vascular movement on stent fatigue performance has not been fully studied.

However, more and more clinical results have revealed that the movement of vascular has an important impact on stent fatigue. Lee et al. [40], Shaikh et al. [41] and Umeda et al. [30] suggested that deformations of the vasculature such as bending, tensile, shear, and torsion, which were caused by heart contractions, lead to alternating stress states in the stent. This alternating state of stress is one of the main reasons for stent fracture. Marrey et al. [33] demonstrated that during the alternation between systolic and diastolic states of the heart, the stent underwent reciprocating conditions, which included stent fatigue and stent fracture. Doi et al. [42] found that during the transition from the systolic to the diastolic state of the heart, the twist of the coronary artery was one of the major reasons for stent fatigue and fracture.

Thus, the vascular movement during the beating of the heart influences the stent mechanical properties (i.e. the fatigue and fracture). Among studies of stent mechanical properties, only the effect of vascular pulsation (VP) caused by heart contractions has been studied, without any consideration for the effect of vascular movement. So it is necessary to determine whether the motion of the artery during heart contractions, negatively impacts stent mechanical performance or not.

The movement of the coronary artery caused by heart contractions is complex, comprised of rigid motion, dynamic twists and dynamic bending. The rigid motion of the coronary artery was neglected because it only changed the position of the stents and artery without changing the stress in the stents or artery. The effect of vessel dynamic twist (VDT) was also ignored accordingly, because anatomical studies proved that coronary arteries undergo important curvature changes throughout the cardiac cycle [43].

In this study, structural FE models were implemented to simulate stent expansion in curved vessel and to investigate the effects of VDB on the long-term mechanical properties of the stent. The aim of this work was to provide a mechanical explanation for the increased risks of stent failure after being implanted in the body. Furthermore, the effect of VDB was explored for its relative importance during mechanical analysis of coronary stents and was to determine whether the motion of the artery during heart contractions, negatively impacts stent mechanical performance or not. The effect of VP on stent mechanical properties was also studied and compared to the effect of VDB.

## Methods

In order to compare the effect of VDB and VP on stent’s mechanical properties, two simulation models were built. The only differences in these two models were the boundary conditions. Details of the simulation models are described as follows.

### Stent model

**Fig. 1:**
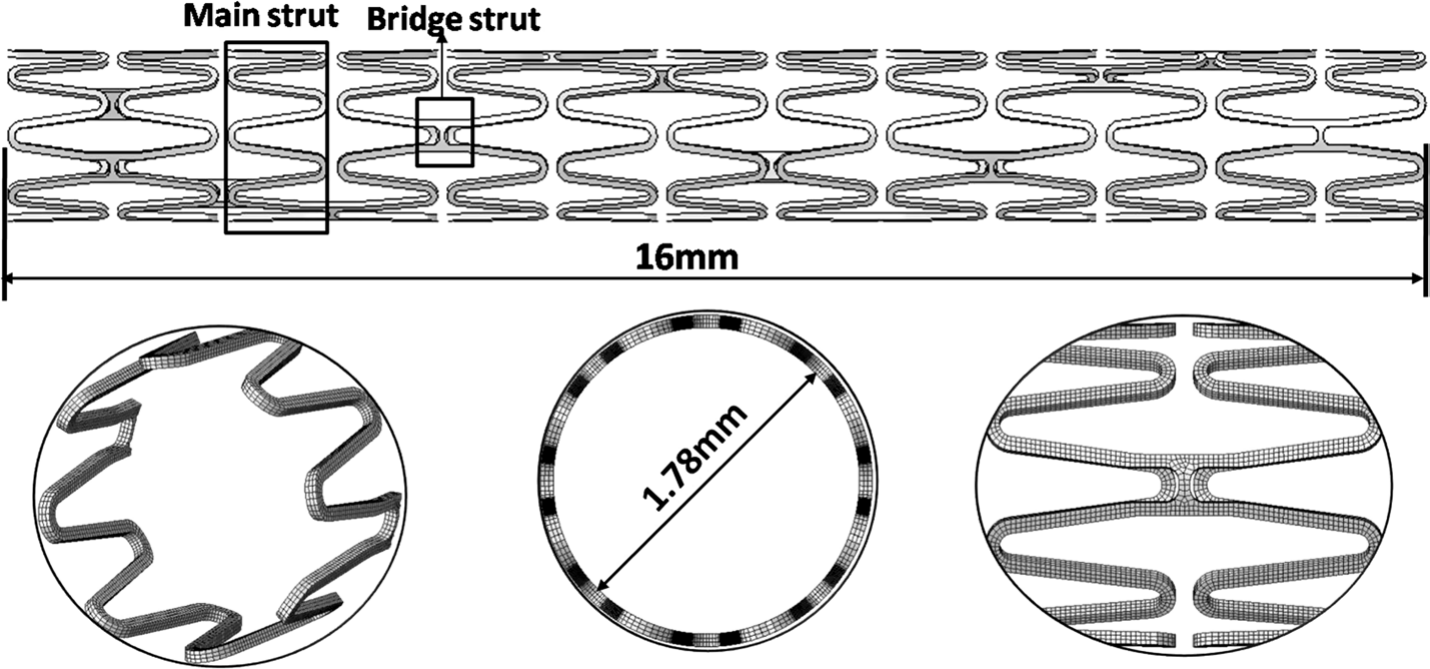
The stent geometry model and finite element mesh

The stent was a typical open-cell design (an Endeavor™ like stent), with 13 loops along the axial direction and 16 struts in each loop. The stent had a length of 16 mm, inner diameter of 1.78 mm, and thickness of 0.08 mm. Eight-node linear element with reduced integration and hourglass control (Abaqus element type C3D8R) was applied to mesh the stent. The total number of elements to mesh the stent was 76,104, which were based on mesh sensitivity studies. Figure 1 shows the geometry model and finite element mesh of the stent.

The material of the stents was the L605 Co-Cr alloy. The material properties of this alloy were modeled in virtue of an elasto-plastic constitutive model with linear isotropic and kinematic hardening. The material properties of the Co alloy are listed in Table 1. The same model has been used in the work of Marrey et al. [33] to study the stent fatigue performance under the effect of VP, the more details see Ref. [33].

**Table 1:** Material properties of Co alloy

Young’s modulus E (GPa)	0.2 % offset yield strength σ_y_ (MPa)	Ultimate tensile strength *S*_*u*_ (MPa)	Fatigue endurance strength *S*_*a*_ (MPa)
243	547	1449	207

### The balloon model

The balloon had a length of 18 mm, diameter of 3 mm, and thickness of 0.05 mm when it was fully deflated. The balloon was an isotropic, linear-elastic material with a Young’s modulus of 900 MPa, a Poisson’s ratio of 0.30 and a density of 2000 kg/m^3^ [44].

The balloon was meshed using 4-node membrane elements with reduced integration and hourglass control (Abaqus element type M3D4R). The total number of elements for the balloon was 12,120 based on mesh sensitivity studies.

**Fig. 2:**
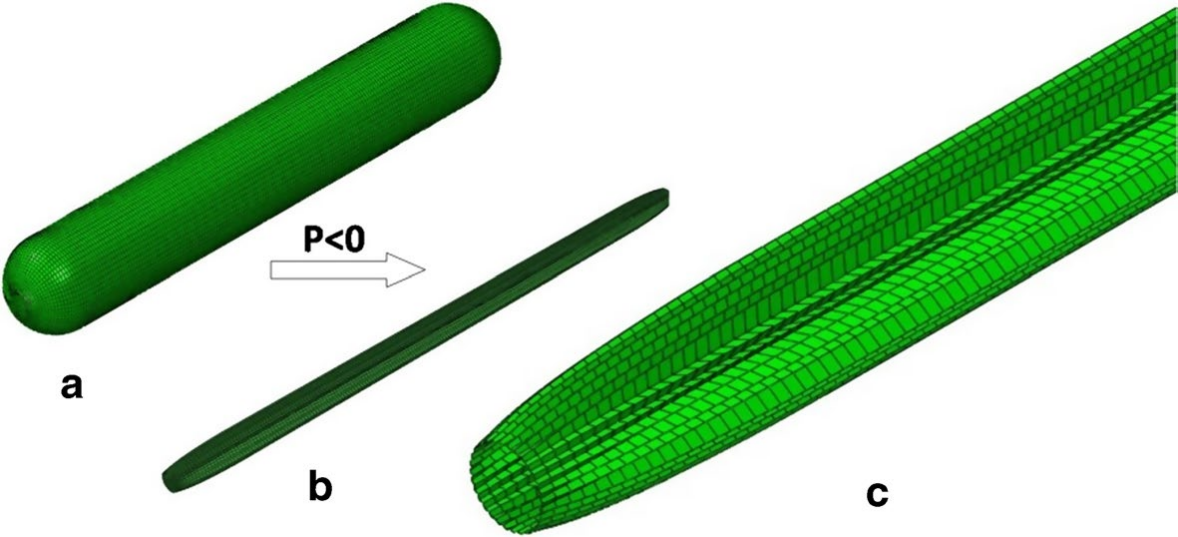
The balloon model of deflation and folding process. **a** is the original unfolded balloon, **b** is the folded balloon, **c** is the details of folded balloon

In this paper, the balloon was compressed by a negative pressure of 0.01 MPa applied on its inner surface with proximal and distal ends fully constrained, as shown in Fig. 2. The balloon was inserted into the stent after deflation.

### Coronary model

**Fig. 3:**
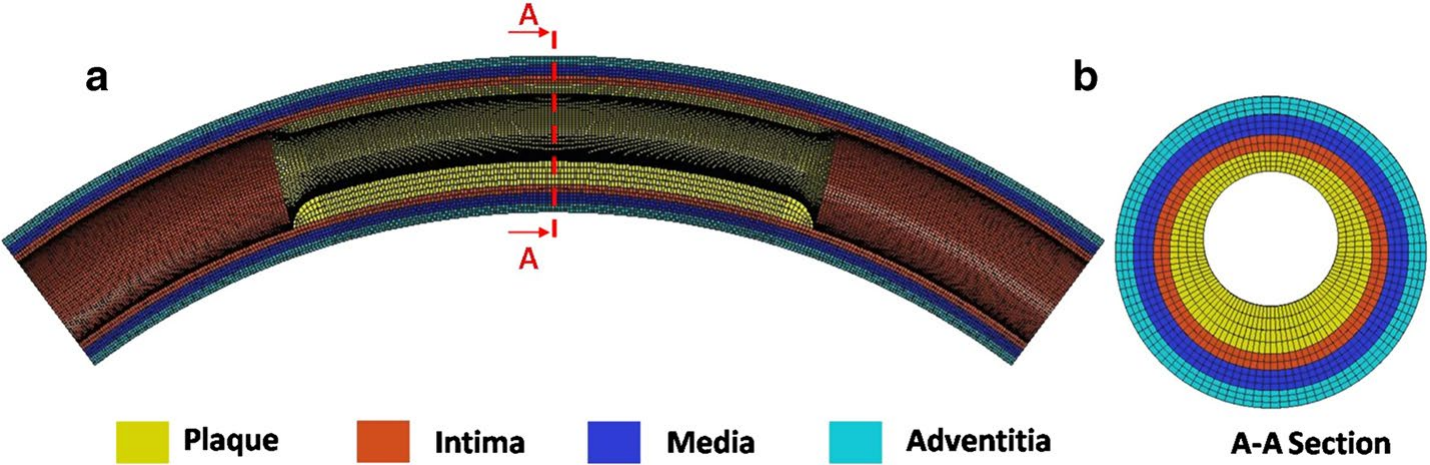
The coronary artery model. **a** is the structure and the finite element mesh of the coronary artery model. **b** is the section and the mesh details of the artery model. The legend indicates the different material regions considered in the model

The coronary artery model was a simplified tube model with a small curvature. The initial curvature radius of the coronary artery is 30 mm [45]. The coronary artery has a length of 30 mm (along the center line), inner diameter of 3.0 mm, and thickness of 0.9 mm. An asymmetric atherosclerotic plaque which has a length of 14 mm is built in the model. The maximal grade of stenosis is set as 60 % of the normal vessel and is located in the middle section of artery. The eight node linear brick, reduced integration elements with hourglass control (Abaqus element type C3D8) was used to mesh the coronary artery. The total number of elements to mesh the stent was 136,360 based on mesh sensitivity studies, as shown in Fig. 3.

The coronary artery consists of three layers: the intima, media, and adventitia, with thicknesses corresponding to 0.28, 0.32, and 0.3 mm respectively. The mechanical behavior of the coronary artery was modeled using a homogeneous, isotropic and hyper-elastic constitutive model, based on the work of Holzapfel et al. [46]. The constitutive law was based on a reduced polynomial strain energy density function, U, of sixth order:

1 $${\text{U}} = \mathop \sum \limits_{i = 1}^{6} {\text{C}}_{i0}\,(\bar{I}_{1} - 3)^{i}$$

Here, $\bar{I}_{1}$ is the first invariant of the Cauchy–Green tensor:

2 $$\bar{I}_{1} = \bar{\lambda }_{1}^{2} + \bar{\lambda }_{2}^{2} + \bar{\lambda }_{3}^{2} ,\begin{array}{*{20}c} {} & {} \\ \end{array} \bar{\lambda }_{i} = J^{( - 1/3)} \lambda_{i}$$

where, *λ*_*i*_ are the principal stretches and *J* is the total volume ratio.

The material parameters for each layer used in this model are listed in Table 2.

**Table 2:** Coefficients of the strain energy density function for each layer of the coronary artery

	C_10_ (MPa)	C_20_ (MPa)	C_30_ (MPa)	C_40_ (MPa)	C_50_ (MPa)	C_60_ (MPa)
Plaque	2.38e−3	1.89e−1	−3.88e−1	3.73	−2.54	5.73e−1
Intima	6.79e−03	5.40e−01	−1.11	10.65	−7.27	1.63
Media	6.52e−03	4.89e−02	9.26e−03	0.76	−0.43	8.69e−02
Adventitia	8.27e−03	1.20e−02	5.20e−01	−5.63	21.44	0.00

The bending of coronary artery was driven by the artery movement. The artery movement was simulated by changing the sphere radius (R), with the center of the sphere fixed at the coordinate origin. The harmonic curvature variation was adopted with the same parameters used in Weydahl et al. [45] and Moore et al. [47]. More specifically, R was expressed as a sinusoidal function:

3 $$\text{R(t)} = \text{R}_{0} \left( {1 \,+\, \updelta \sin \left( {\frac{\uppi \text{t}}{\text{T}}} \right)} \right)$$

where the mean sphere radius, R_0_ was set as 30 mm, parameter δ was 0.15, and T as 0.75 s.

**Fig. 4:**
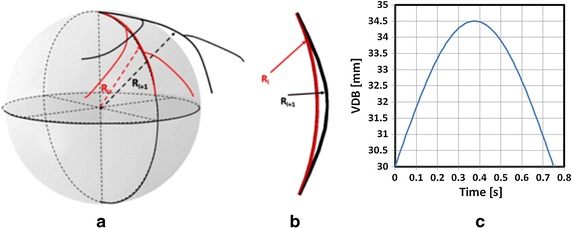
The schematic diagram of the coronary artery movement and the motion model. **a** is the schematic diagram of coronary artery motion caused by the heart; **b** is the simplified VDB model; **c** is the dynamic bending of the vessel

Figure 4a shows the schematic diagram of the coronary artery motion caused by the heart beating. In this model the heart beating was simulated by changing the sphere radius. Figure 4b shows the simplified VDB model. Figure 4c shows the fluctuation of the mean sphere radius according to Eq. (3).

### Numerical model

In this work, the simulation as two major mechanical processes: the stent expansion in a curved vessel and the stent working in the curved vessel (the stent under the effect of VDB or VP). The stent expansion process was simulated using the explicit dynamics method (Abaqus/Explicit) and the second one was simulated using the implicit method (Abaqus/Standard). A data (mainly include the stresses of stent and artery after stent implanted) transfer process was carried out between two different mechanical processes, which is referred to as a typical explicit-implicit continuous analysis method.

In order to compare the effect of vascular dynamic curvature with that of vascular pulsation, two different simulation models were carried out: Model-1 was the VDB effect simulation and Model-2 was the VP effect simulation.

**Fig. 5:**
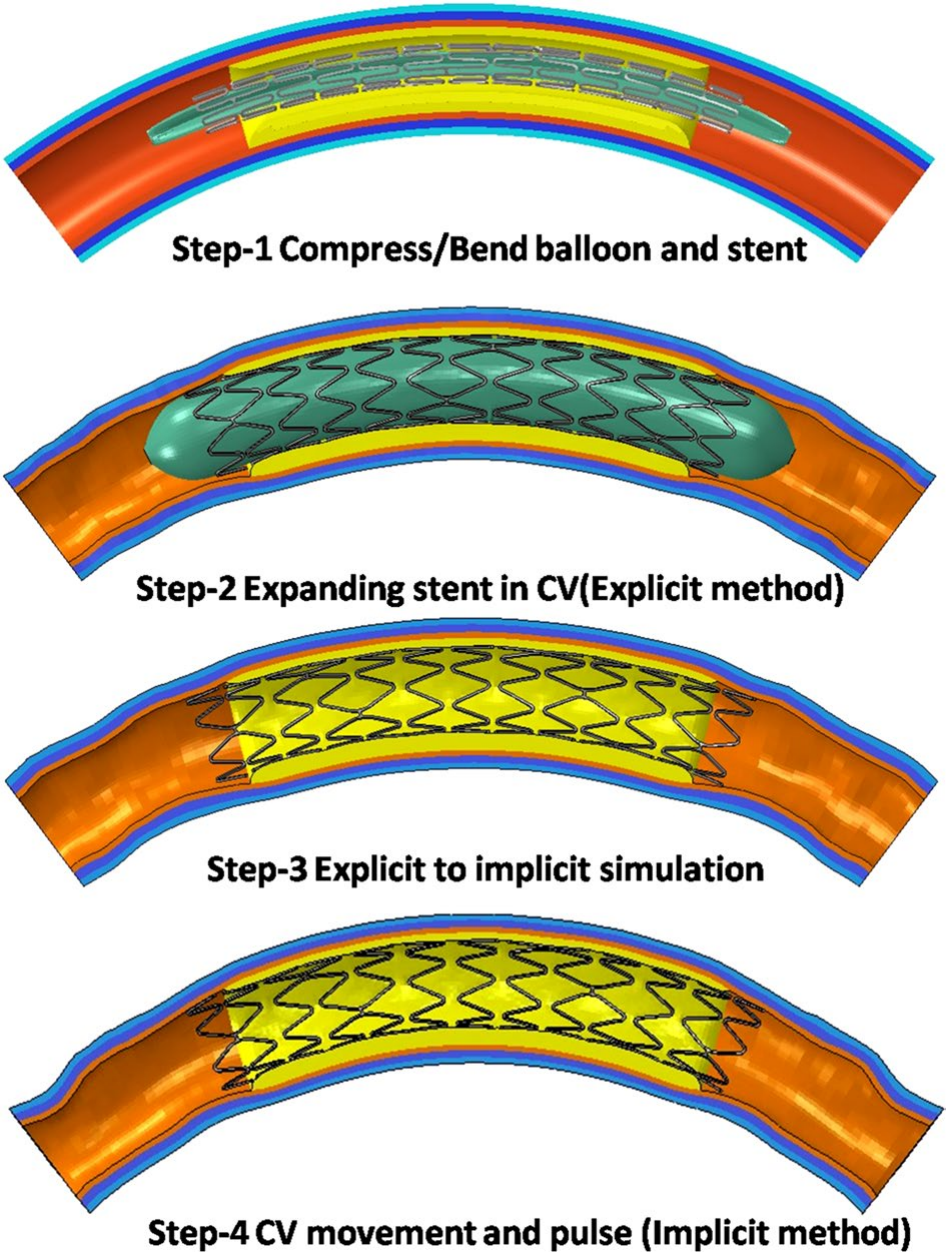
Simulation steps and boundary conditions

The simulation was divided into four steps in each model, as shown in Fig. 5.

The details of each step are defined below for Model-1.

#### Step1: Compressing the stent to the folded balloon and bending stent and folded balloon

This step was a pre-treatment step which include two process: compressing the stent to the folded balloon and bending stent and folded balloon to meet the initial curvature of the artery. For the compressing stent process, the balloon was constrained; two ends of the stent were constrained in the circumferential direction and a radial displacement of 0.3 mm was applied on the outer surface of the stent to compress it. After being released, the radial displacement of outer and inner stent was about 1.3 mm. For the bending stent process, the stent and balloon was free, a rigid catheter was imposed in the outer surface of stent and balloon, the bending deformation of stent and balloon is driven by the configure change of the rigid catheter set by the displacement boundary conditions. This method for bending stent and balloon had been applied to study stent’s mechanical properties by F. Auricchio et al. [48].

#### Step2: Expansion of the stent in the curved vessel and release of the pressure applied on balloon

In this step, two processes were simulated: expansion of the stent in the curved vessel followed by the release of the pressure. A load of 1.0 MPa was initially applied on the inner surface of balloon for expansion. When the stent was completely expanded under a full load of 1.0 MPa, the load was gradually decreased from 1.0 to 0 MPa. Two ends of the balloon and coronary artery were constrained in all directions and two ends of the stent were constrained in the circumferential direction.

Due to the high nonlinear compression/expansion of the balloon and stent in Step1 and Step2, a quasi-static analysis was performed using Abaqus/Explicit. In order to control the ratio of kinetic energy to the total strain energy under 5 % in each step, the simulation time was set as 5 s in Step1 and 13 s in Step2.

#### Step3: Transferring mechanical information from Abaqus/Explicit to Abaqus/Standard

When the load applied on the inner surface of balloon was released completely, the mechanical information (i.e. displacement U, stress σ) of the stent and the artery was transferred from the Abaqus/Explicit module to the Abaqus/Standard module. The residual stresses after stent expansion were being considered to analyze stent’s mechanical properties under the effect VDB or VP. The transferred information would be set as the initial conditions of Step4. In this step, the balloon was withdrawn from the simulation model. The boundary conditions of the stent and coronary artery were the same as in Step2.

#### Step4: Applying the VDB on the stent

A spatial displacement function described in section “Coronary model” was applied to the outer surface of the artery to simulate its bending. The stent was set free in all directions.

For Model-2, the boundary conditions in Step1, Step2 and Step3 were the same as those in Model-1, with the only differences occurring in Step4 of the two modules. In Step4, different values of cyclic pressure ranging from 80 mmHg (diastolic) to 120 mmHg (systolic) were applied on the inner surface of the stent to simulate the pulsation of vessels caused by the beating of the heart. The load was regarded as harmonic, with the period of the load being 0.75 s, which was equal to the cardiac cycle. It was considered to be the same as the load in the work by Zhi et al. [35]. The two ends of the artery were constrained in all directions and the stent was set free in all directions.

In this work, a surface to surface contact algorithm is selected to model the nonlinear contacts in Step1, Step2 and Step4. The coulomb friction model is used to model the frictional contacts between the balloon and stent, balloon and artery as well as the stent and artery. This contact method was used in many of the works about the interactions between stents and vessels [8, 11, 17, 19–21, 23, 25]. A value of 0.1 was selected in the whole simulation [19, 20].

### The explicit-implicit coupling method

In this work, an explicit-implicit coupling method was used, the key step of this approach was coupling the Abaqus/Explicit module and the Abaqus/Standard module. This step mainly include two aspects:

1. The deformed configure of stent and artery at the end time of stent expansion process in Explicit module was imported to the Standard module and it was set as the initial configure of the stent and artery for analyzing stent response under VDB and VP.
2. The residua stresses of stent and artery (the balloon had been removed from the simulation model) after stent implanted was set as the initial stress conditions of the implicit simulation. The plastic strain of stent during the expansion process was omitted due to under the effect of VDB or VP the mechanical properties of stent were in the elastic state.

### Post-processing

The predicted effective alternate stresses and effective mean stresses were then used to calculate a fatigue safety factor (FSF) distribution by utilizing the modified-Goodman relationship, as used by Marrey et al. [33]. The FSF, which essentially quantifies the proximity of the effective mean stresses and effective alternate stresses at any given numerical integration point to the limiting Goodman curve (Fig. 14), was determined through:

4 $$\frac{1}{FSF} = \frac{{\sigma_{m} }}{{S_{u} }} + \frac{{\sigma_{a} }}{{S_{a} }}$$

Here, *σ*_*m*_ is the effective mean stress, *σ*_*a*_ is the effective alternate stress, *S*_*u*_ is the ultimate stress and *S*_*a*_ is the endurance limit for zero mean stress. Associated with the ultimate tensile strength and endurance strength of the Co alloy stent material, respectively; actual values used in the current analysis are listed in Table 2.

After collecting the values of principal stresses (*σ*_1_, *σ*_2_ and *σ*_3_) at each node, the effective mean and alternate stresses were calculated using the following equations:

5 $$\sigma_{m} = \frac{1}{\sqrt 2 }\sqrt {(\sigma_{1m} - \sigma_{2m} )^{2} + (\sigma_{2m} - \sigma_{3m} )^{2} + (\sigma_{3m} - \sigma_{1m} )^{2} }$$

6 $$\sigma_{a} = \frac{1}{\sqrt 2 }\sqrt {(\sigma_{1a} - \sigma_{2a} )^{2} + (\sigma_{2a} - \sigma_{3a} )^{2} + (\sigma_{3a} - \sigma_{1a} )^{2} }$$

where the *σ*_1*m*_, *σ*_2*m*_ and *σ*_3*m*_ are principal mean stresses, while the *σ*_1*a*_, *σ*_2*a*_ and *σ*_3*a*_ are principal alternate stresses. These values were used to build Goodman diagrams that are commonly used to quantify the combined effect of mean and alternating stresses on the fatigue life of a material.

## Results

### The validity of simulation results

**Fig. 6:**
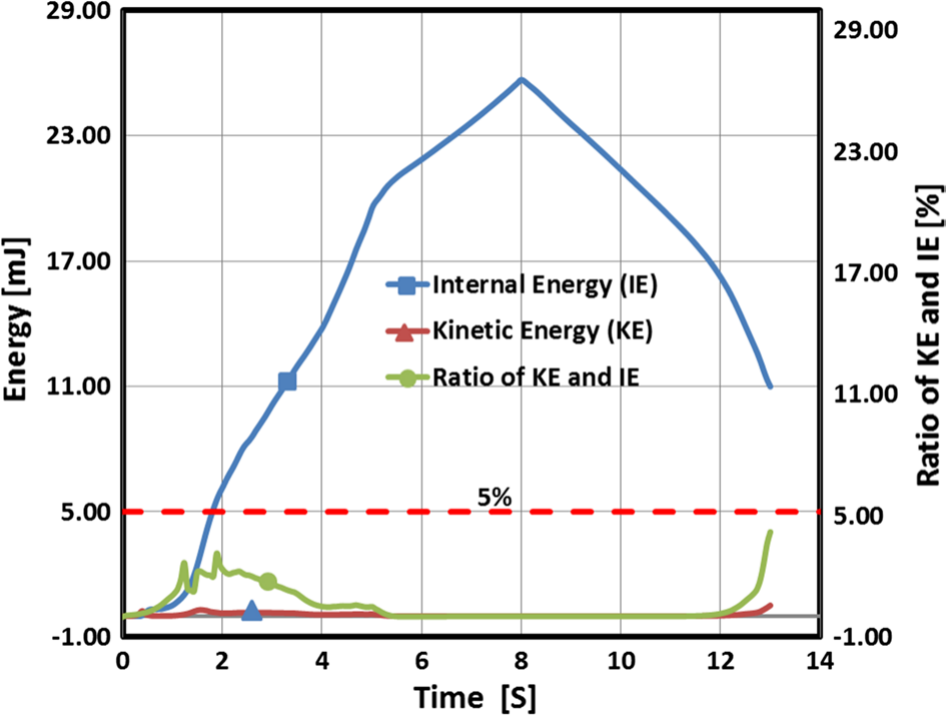
Time history of the internal and kinetic energy during the stent expansion process

To evaluate the simulation with the explicit method, the total kinetic and total internal energy of the system was used to judge whether the results were valid. It was a quasi-static simulation method, according to the Abaqus suggestions, when the kinetic energy accounted for less than 5 % of the internal energy, the results were acceptable [23, 26]. The internal energy and kinetic energy in respect to the time during stent expansion is shown in Fig. 6. The ratio of kinetic energy to internal energy was less than 5 %. Thus, the result of the expanding stent in the curved vessel by explicit method was acceptable.

### The deformation and stress of vessel under the effect of VDB

**Fig. 7:**
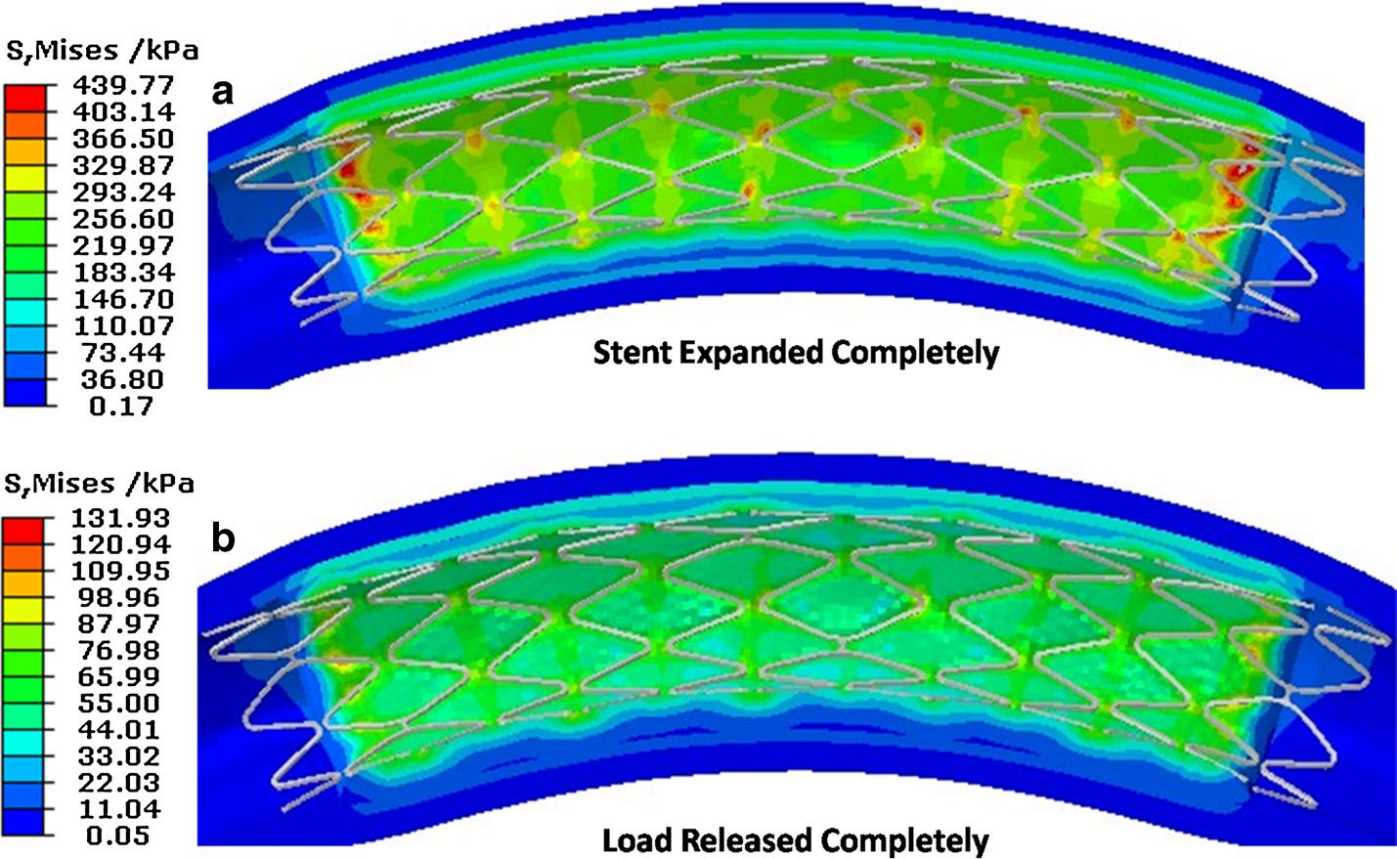
The shape of the stent and stress distribution on the vessel during stent expansion. **a** is the stent completely expanded, **b** is the load completely released

The shape of the stent in the curved vessel and stress distribution of the vessel at two different instantaneous times during the stent expansion process are shown in Fig. 7. When the stent was expanded completely, the maximal stress of the artery was 439.77 kPa and was localized in the zone which was close to the distal/proximal end of the plaque. The peak stress in the curved vessel reached 131.93 kPa after the load was released completely and appeared on the vessel zone which was close to the ends of plaque. During the stent expansion process, the maximal compression effect of the stent on the artery appeared at two ends of the plaque.

**Fig. 8:**
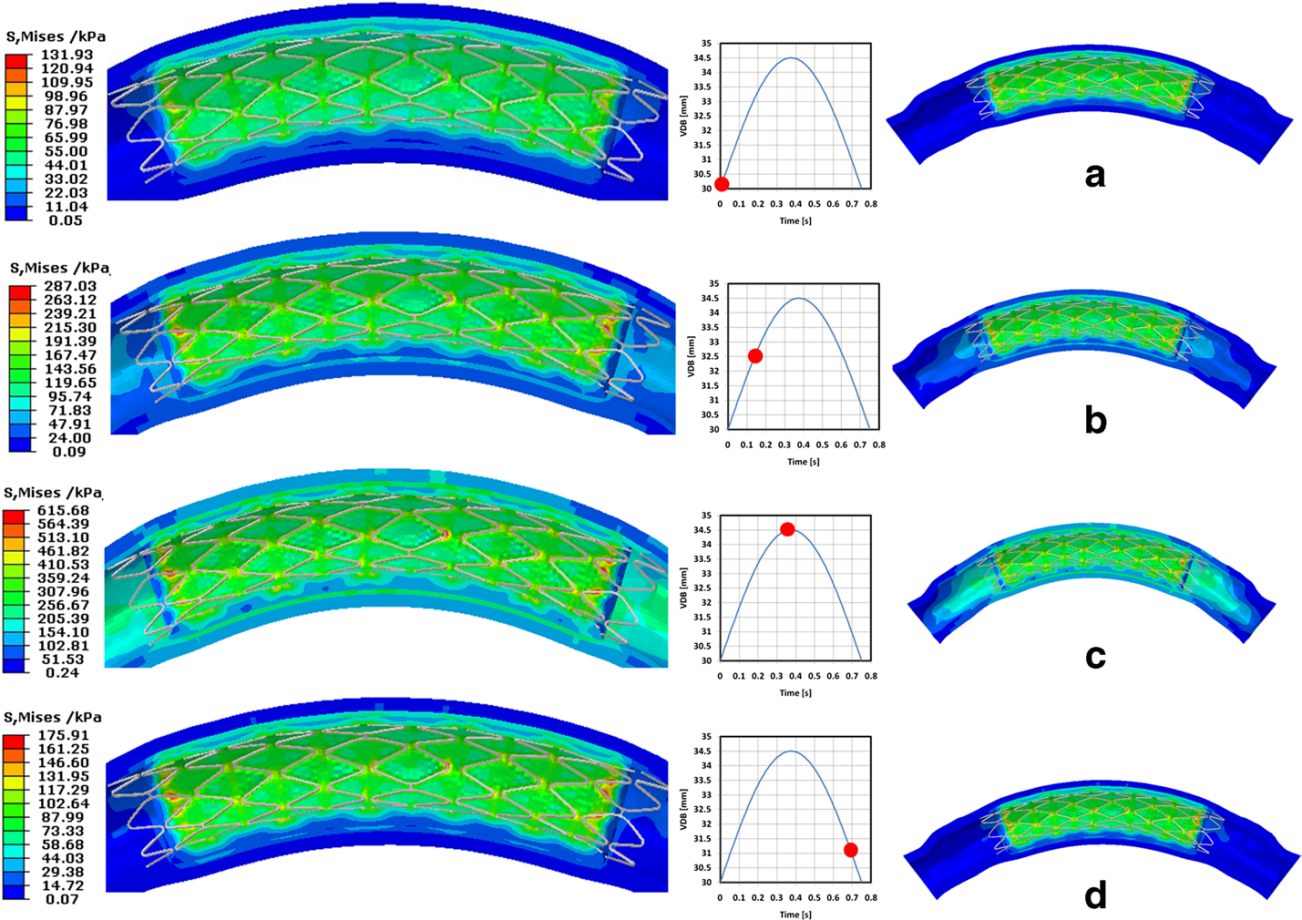
Stress distribution in the vessel during artery moving process. **a** is the initial time, **b** is a quarter of a cardiac cycle, **c** is half of a cardiac cycle, **d** is 4/5 of a cardiac cycle

The map of the stress distribution in the curved vessel at four different instances during the vascular dynamic curvature in a cardiac cycle is shown in Fig. 8. The initial mechanical properties of the stent and artery were the same as those reported in Step2 (as shown in Fig. 7b). This suggests that the mechanical information (the stress of vessel after stent implanted) in Step2 was correctly transferred to the standard analysis module in Step4, as initial conditions. At a quarter of the cardiac cycle, the maximal displacement of the curved vessel was 0.6 mm, while the maximal stress of the vessel was 287.03 kPa which was localized in the two ends of plaque. At half of a cardiac cycle, the displacement of the vessel reached a maximal value of 1.8 mm with the maximal stress of the vessel being 615.68 kPa which was localized in the middle position of vessel. At 4/5 of a cardiac the maximal stress of the vessel was 175.91 kPa. The stress distributed in the vessel during the process of vessel movement was greater than that which occurred during the stent expansion process.

### The bending deformation of stent under the effect of VDB

**Fig. 9:**
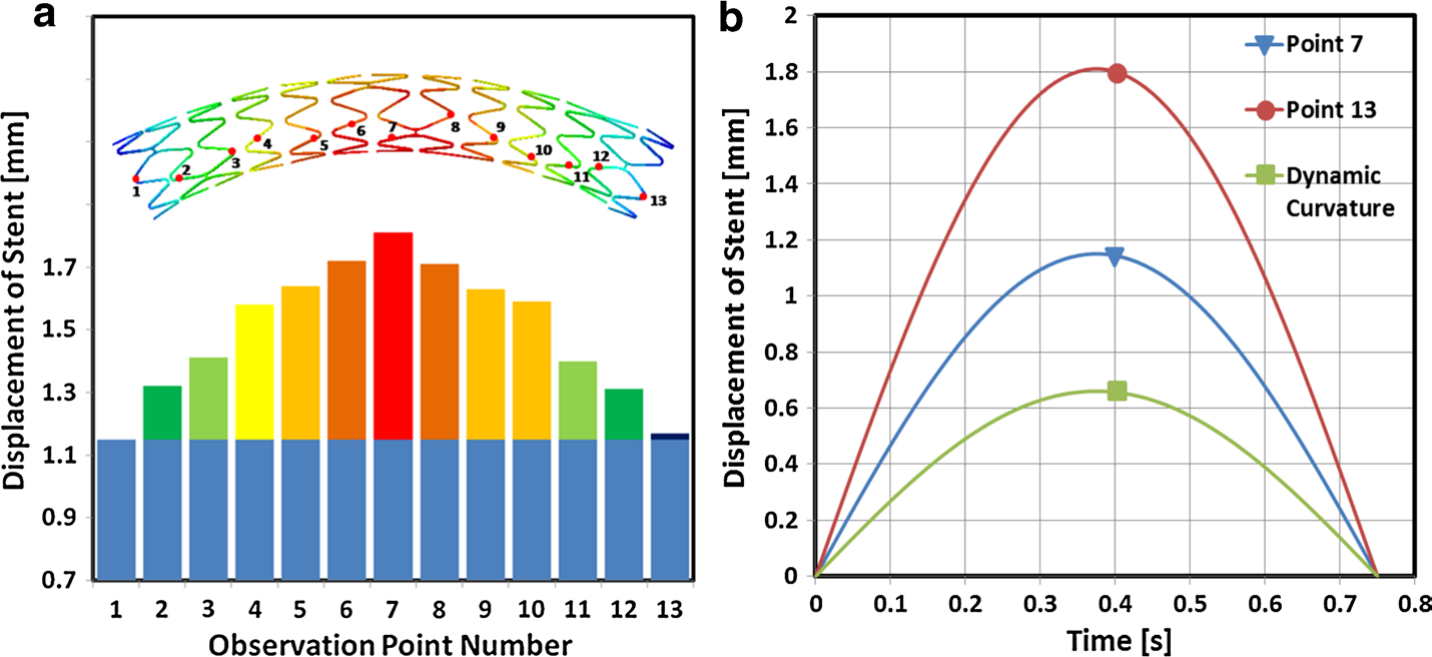
The displacement of the stent. **a** The map of the stent displacement at half of a cardiac cycle during the process of vascular motion. **b** The displacement of the stent in relation to time, at two positions

At half of a cardiac cycle, the map of the vascular displacement is shown in Fig. 9a. From the map, the obtained displacement at every loop was approximated. Along the axial direction of the stent, 13 typical positions were marked out at every loop of the stent (shown in Fig. 9a as the observation Points 1–13) to measure the stent’s displacement during the vascular dynamic curvature process (as shown in Fig. 9a). The maximal displacement was 1.8 mm, which occurred at the middle loop of the stent and the minimal displacement was about 1.1 mm which occurred at the two end loops of the stent. The displacement of the stent was symmetrical about the middle position. The displacement of the stent included two parts: the rigid motion and the bending deformation. The displacement difference between these 13 points can be used to describe the bending deformation of the stent (as shown in Fig. 9a). The maximal bending deformation of the stent was about 0.65 mm at half of a cardiac cycle. The bending deformation of the stent was symmetrical about the middle position which was a typical simplified deformation model of a support beam.

The displacement of Point 13 and Point 7 relative to the cardiac cycle is shown in Fig. 9b. The displacement difference between Point 13 and Point 7 was caused by the dynamic curvature of the stent during the process of VDB. The maximal bending deformation of the stent occurred at half of a cardiac cycle. During the process of VDB, the stent was in a state of dynamic bending deformation. Under this dynamic bending deformation state, the stent experienced alternating stress states, which would cause the fatigue and failure of the stent.

### The stress of stent during the process of VDB

**Fig. 10:**
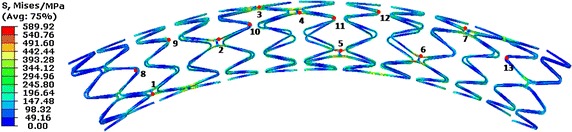
The distribution of stress in the stent at half of a cardiac cycle

Figure 10 shows the von-Mises stress distribution of the stent at half of a cardiac cycle. The maximal stress of the stent was 589.92 MPa, which occurred in the bridge struts of the stent. In the axial direction, the stress mainly distributed in the middle loops of the stent; corresponding to the maximal bending deformation. Also, the stress of the bridge struts was greater than that of the main struts in the circumferential direction. 13 observation points were marked in the stent, Points 1–7 were located at the bridge struts and Points 8–13 were located in the ends of the stent loops in order to observe the stress of the stent relative to time.

**Fig. 11:**
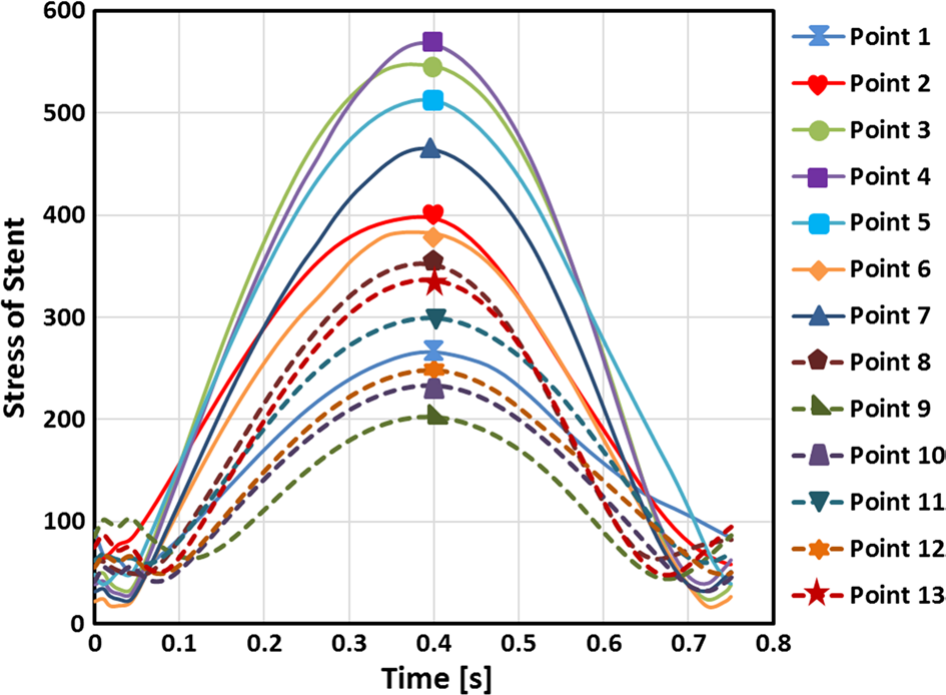
The stress of the stent in terms of time. Points 1–7 were located at the bridge struts and Points 8–13 were located in the ends of the stent loops

Figure 11 shows the stress of the stent’s marked points in terms of time, during the process of VDB. It shows that the stent bears a cyclic stress due to the vascular dynamic curvature. Also, the relationship of the stress and time can be expressed by a sine wave function as time changes. Figure 11 shows the stress in the bridge struts of the stent (Points 1–7, marked in Fig. 10) and the stress in the main struts (Points 8–13, marked in Fig. 10). In the axial direction, the stress mainly occurred in the middle loops where the bending deformation was higher, (i.e. the stresses at Points 3, 4, and 5 were greater than at Points 1, 2, 6 and 7). It was observed that the stress mainly occurred in the bridge struts, with the maximal stress of 589.92 MPa being located in bridge struts, while the maximal stress in the main struts was only 380 MPa. The reason for the aforementioned occurrence is that the stress of the stent is mainly caused by the bending deformation in the VDB process. While in the bending deformation state, the stent’s stress mainly occurred in the bridge struts. From the view of time, the maximal stress occurred at half of a cardiac cycle, when the maximal bending deformation occurred. This also indicates that failure frequently occurs in the bridge struts, because the greater stress causes more severe damages.

## Discussions

### The safety of the stent

In the process of VDB, the stent experienced dynamic bending deformation; the stress placed on the stent was an alternating stress. This was a main contributing factor to stent fatigue. As discussed in the introduction, the VP was another main trigger for stent fatigue which has been considered in previous studies. As a result, the effect of VP on stent fatigue performance was also studied and compared to the effect of VDB.

**Fig. 12:**
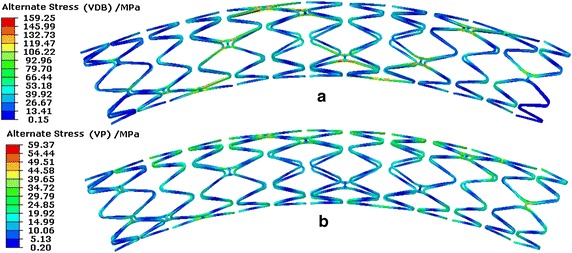
Stent effective alternate stresses distribution of a stent in a cardiac cycle. **a** Distribution with regards to the effect of VDB. **b** Distribution with regards to the effect of VP

Figure 12 shows the stent effective alternate stresses distribution. In regards to the effect of VDB, the stent’s maximal effective alternate stress was 159.25 MPa in a cardiac cycle while only 59.37 MPa with regards to VP. Considering the effects of VP, the effective alternate stress of 48.02 MPa was close to the result obtained by Marrey et al. [33], which was 52 MPa. The maximal deformation of the stent was close to 6 % of the inner diameter of vessel (here it was close to 0.18 mm) in regards to VP, while it was about 0.65 mm (as shown in Fig. 9) in regards to VDB.

The maximal effective alternate stresses occurred in bridge struts as the result of both the VDB and VP. A concentration in the middle loops of the stent was observed where the bending deformation was greater than that in other positions. Also, the effective alternate stress in the bridge struts was higher than that in the main struts with regards to VDB. In terms of VP, the effective alternate stress uniformly distributed along the stent axially and the effective alternate stresses in the bridge struts was similar to that in the main struts. This occurred because the dynamic bending deformation appeared in the stent under the effect of VDB, therefore the main bearing load portion was the bridge struts. Also in terms of VP, the load was uniformly applied on the inner surface of the stent, the deformation of the stent was in the state of dynamic expansion and recoil, therefore the effective alternate stresses in the bridge struts was similar to that in the main struts.

**Fig. 13:**
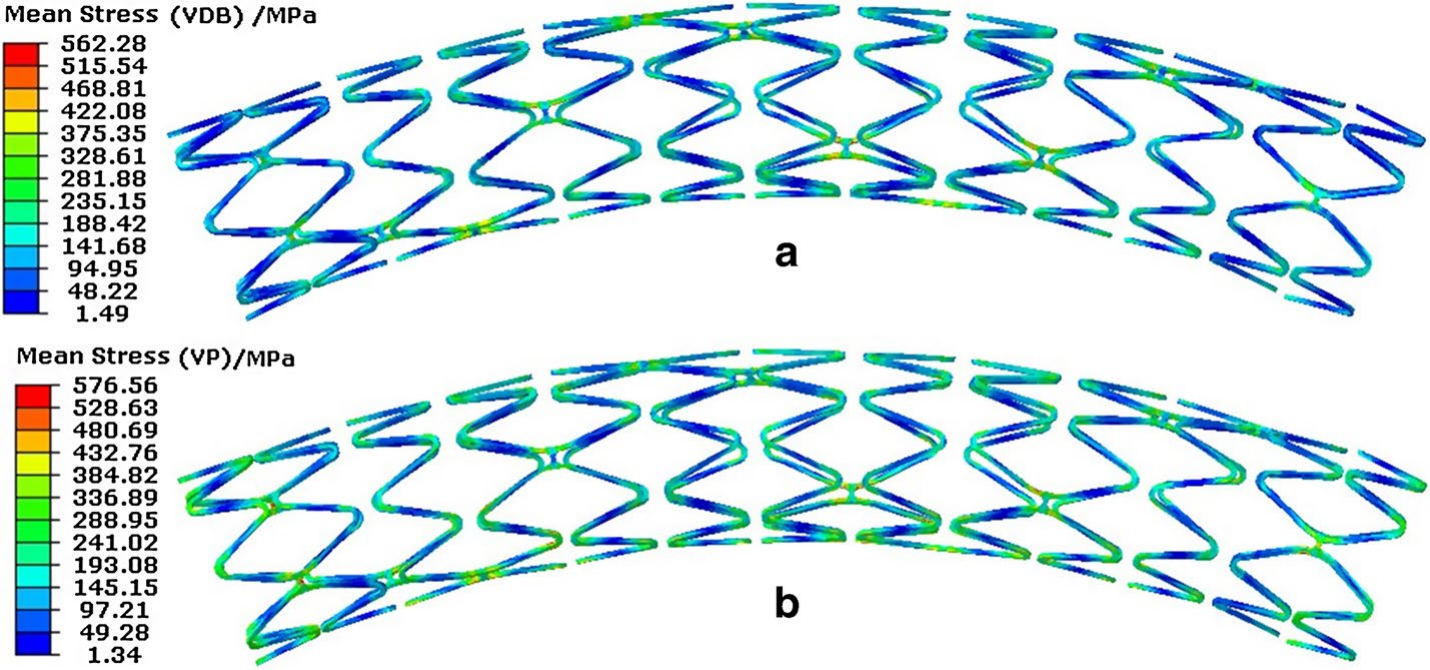
Effective mean stress distribution in the stent. **a** Distribution for VDB. **b** Distribution for VP

Figure 13 shows the distribution of the stent effective mean stresses in a cardiac cycle. In regards to the effects of both VDB and VP, the maximal effective mean stress of the stent was similar according to Fig. 13. With regards to the effect of VDB, the effective mean stress mainly occurred in middle loops of the stent along the axial direction. Along the circumferential direction, the effective mean stress mainly occurred in bridge struts. The stress was uniformly distributed along the stent in the axial direction when regarding VP. This distribution was the same as the distribution of the effective alternate stresses.

**Fig. 14:**
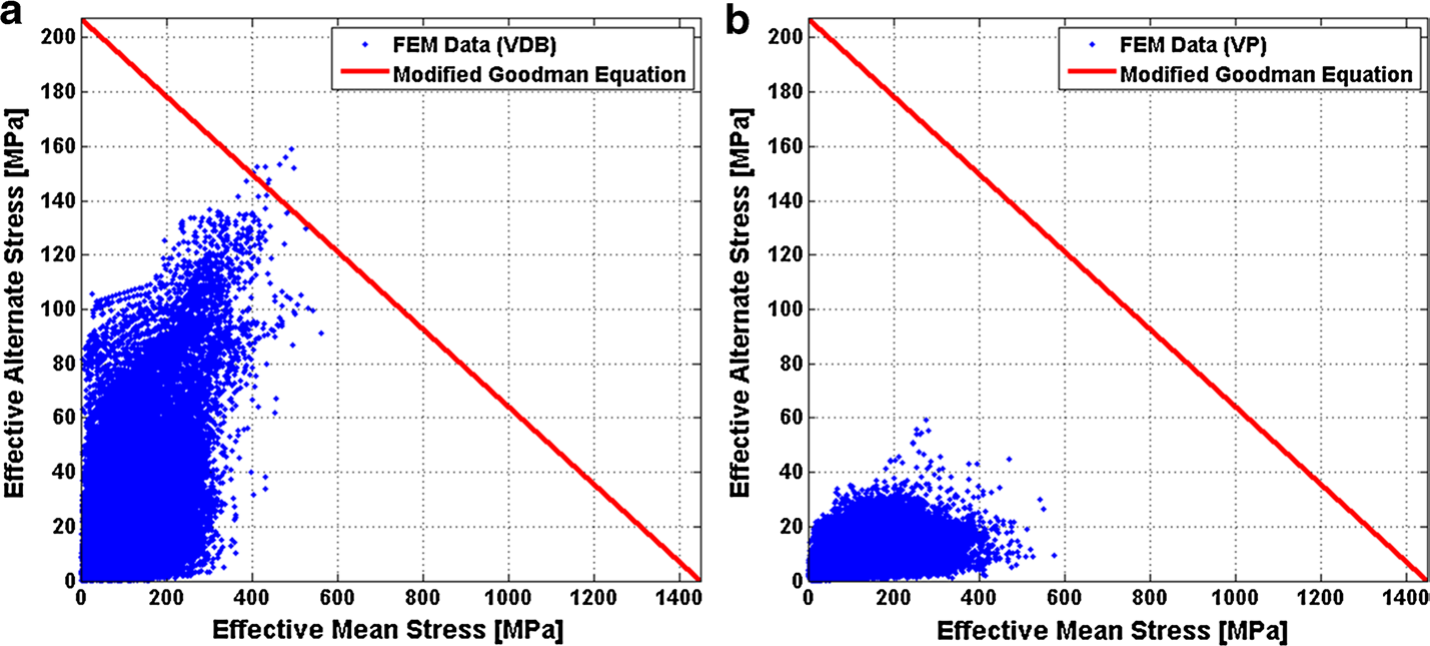
Stent Goodman diagrams. **a** With regards to the VDB. **b** With regards to the VP

Figure 14 shows the Goodman diagram of the stent under two different fatigue loads [(a) with regards to VDB and (b) with regards to VP]. FEM calculated data was below the Goodman diagram failure line, indicating that the stent was able to pass the fatigue life of 4 × 10^8^ cycles under both fatigue loading conditions. These results fitted favorably with those results reported in the literature [30, 33]. When comparing two Goodman diagrams under different loads, the effect of VDB on the stent’s FSF was lower than that caused by VP. As shown in Fig. 14, the FEM results of the VDB were closer to the Goodman curve than those of the VP.

**Fig. 15:**
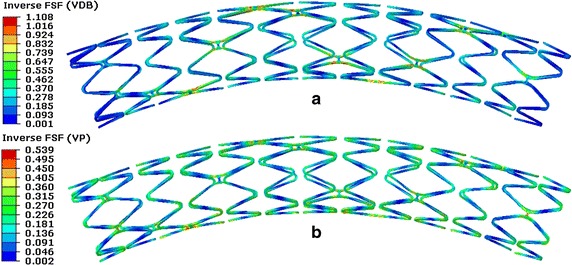
Contour plot of the inverse FSF of the stent. **a** With regards to VDB. **b** With regards to VP

A contour plot of the inverse FSF is shown in Fig. 15. The maximal inverse FSF of the stent for the VDB was 1.108, while it was only 0.539 for the VP. This suggests that the fatigue failure of the stent in regards to the effect of VDB occurred more easily than those regarding VP (if the inverse FSF equals 1, the stent failure occurs). This mainly occurs because, the stent was under higher alternating stress in regards to VDB when compared to that in VP (the effective alternate stress for the VDB was 159.25 vs. 59.37 MPa for the VP). Under two fatigue loading conditions, the predicted worst-case fatigue locations both occurred in the bridge struts. This suggested the fracture of the stent is more likely to happen in the bridge struts, which matched with the results reported in the literature [21], in which the stent fracture was confirmed by medical images in clinic as well as in the literature [49]. So in the analysis model of the stent fracture, the effect of VDB should be considered and it has also been proven useful to optimize bridge struts because the predicted position of stent fracture mainly occurred in the bridge struts from the simulation results and clinical results.

### The impact of the stent on the vessel

**Fig. 16:**
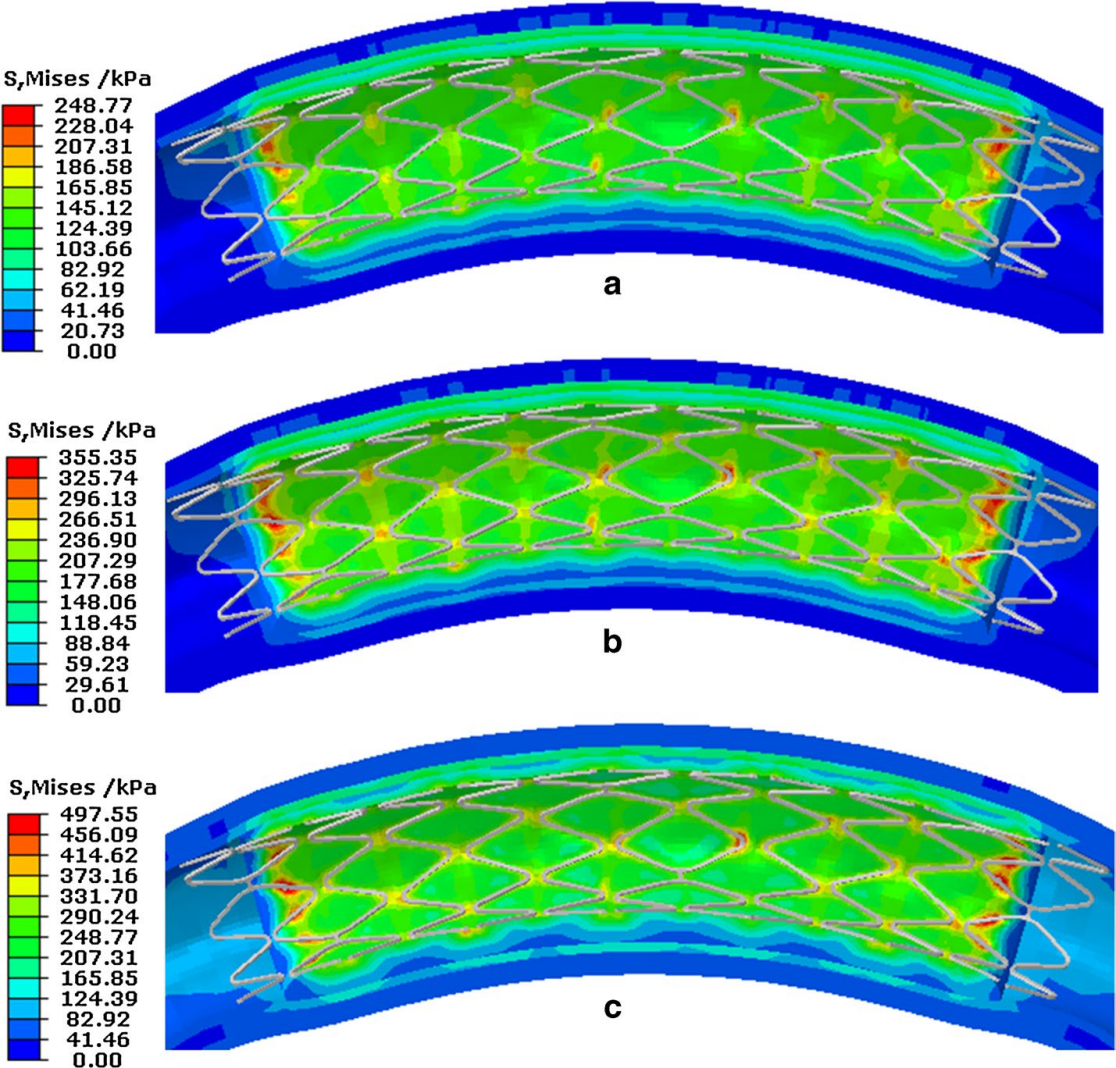
The effective mean stress distribution of vessels caused by the stent. **a** During stent expansion. **b** Distribution caused by VP. **c** Distribution caused by VDB

Currently, the reason for ISR is not yet been clearly understood. Clinically it is generally accepted that the vessel injury is caused when the stent presses against the vessel wall after stent implantation. Based on the fact that ISR is an indicator of vessel injury, it is useful to study IRS factors in order to predict the stress of the vessel caused by the stent. The FEM has been used as the basis for studying IRS factors [21–28]. Figure 16 shows the effective mean stress of the vessel during different stent service processes [(a) shows the process of stent implantation, (b) shows the effect of VP in a cardiac cycle, and (c) shows the effect of VDB in a cardiac cycle].

During the process of stent implantation, the maximal effective mean stress of the vessel caused by the stent was 248.77 kPa. Considering VP, the maximal stress of the vessel caused by the stent was 355.35 kPa. Considering VDB, the maximal stress of the vessel caused by the stent was 497.55 kPa. The stress level of the vessel caused by the stent was higher under VDB than that under VP or during stent expansion; this suggested that more injury occurred as a result of VDB. It indicated that in the analysis model of stent’s mechanical properties, the effect of VDB caused by the beating heart should be considered.

### Discussion of the simulation method used

For the previous studies on stent mechanical properties, the implicit simulation method was used more widely in early works [4–7, 13–19]. However, only simplified models could be solved using this method because it was difficult to solve the highly nonlinear problem [8]. Since 2008, the explicit method was more and more widely used [9–12, 21–28] in most studies about stent mechanical properties. Up until now, a combination of explicit and implicit methods has never been utilized to analyze the stent mechanical properties.

In fact, the explicit method was a dynamic solution method and the results included inertial effect and kinetic energy, while the static analysis was contrary to it. If the explicit method was applied to solve static problem (the quasi static problem), it was necessary to reduce effect of the kinetic energy of system on the simulation results. A common strategy in previous studies was to increase the loading time and to achieve the purpose of reducing the kinetic energy. At the same time the simulation time also increased with the increase of loading time.

In addition, the stable increment time of the explicit method is.very small after the stent has been meshed. Based on this increment time, a long simulation time is needed. Instead, a mass scale method is used to artificially increase the stable time, however the kinetic energy of the system must also correspondingly be artificially increased. It was very important to find a balance between the kinetic energy of system and the simulation time. But for the implicit method, there was not this limitation. So using the implicit method the simulation time could be saved and the results were more reliable than that using the explicit method.

Here, a novel way to simulate a stent’s mechanical responses was provided. The stent expansion process was simulated using the explicit dynamics method (Abaqus/Explicit), due to the fact that the problem was highly nonlinear. The stent mechanical properties under the effect of VDB and VP, were simulated using the implicit method (Abaqus/Standard) to save computational time. A data transfer process was carried out between two different mechanical processes, which is referred to as a typical explicit-implicit continuous analysis method. From the simulation results, it was concluded that this method can be applied to study stent’s mechanical properties. In this work, we describe the explicit-implicit continuous analysis method in order to simulate the stent’s mechanical properties with the application of this method on other problems to be explored in the future.

## Conclusion

In this work, an explicit-implicit coupling finite element simulation was employed to investigate the coronary stent’s mechanical properties considering the effect of VDB after the stent was expanded in a curved vessel. Compared with the mechanical properties of a coronary stent with regards to VP, the following conclusion can be drawn.

Under the effect of VDB, the predicted worst case of stent fracture was mainly located in the bridge struts of the middle loops, which matches well with the results reported in the literature in which the stent fracture was confirmed by medical images. For the effect of VP on the stent, the predicted worst-case of stent fracture was also located in the bridge struts rather than in the middle loops. The result for the effect of VDB was consistent with clinical observations. The effect of VDB was a reason for the increased risk of long-term stent fracture.

Under the effect of VDB, the stent was in a bending deformation state, where the deformation of the stent under VDB was larger than that under VP, which caused a higher alternating stress level under VDB (the effective alternate stresses were 159.25 MPa under VDB vs. 59.37 under VP). The fatigue failure of the stent under VDB occurred more easily than that under VP (the fatigue safety factor of the stent was lower for the VDB than for the VP, which is mainly caused by the higher alternating stress induced by the VDB). Under VDB, the stress in the vessel was greater than that under VP. During stent expansion, it was suggested that more vascular injury occurs as a result of VDB. The effect of VDB during the beating of the heart would negatively impact stent mechanical performance. Thus, in the analysis model for the coronary stent, the mechanical performance should be considered.

Meanwhile an explicit-implicit coupling method was employed to analyze the long term stent mechanical properties. The results showed that this method is feasible and effective to study stent mechanical properties.
